# Walking ability in adults with severe hemophilia: A cross-sectional study

**DOI:** 10.46989/001c.94374

**Published:** 2024-03-03

**Authors:** Malika Jhandai, Dimple Choudhry, Sudhir Kumar Atri, Pankaj Bhardwaj, Kusum Yadav

**Affiliations:** 1 College of Physiotherapy Pandit Bhagwat Dayal Sharma University of Health Sciences; 2 Medicine Department Pandit Bhagwat Dayal Sharma University of Health Sciences; 3 Department of Physiotherapy Jagannath University

**Keywords:** Hemophilia, Hemophilic arthropathy, Knee arthropathy, 50- Meter walk test, Walking ability

## Abstract

A lack of factor VIII (FVIII) or factor IX (FIX) results in hemophilia, a blood-clotting illness. The mode of inheritance is chromosome X-linked and recessive. The primary symptom of severe hemophilia is spontaneous and recurrent bleeding into joints, muscles, and soft tissues. Unpreventable bleeding may cause arthropathy, chronic discomfort, and muscular atrophy. Therefore, joints’ functional loss affects the functional and walking ability. The aim of this study was to determine the walking ability by measuring the 50-m walk test time in severe hemophilic patients, as compared to the normal population. Sixty subjects (males) in the 18-30 year age group were selected and comprised 30 hemophiliacs and 30 in a control group. The 50-m walking ability was measured in seconds. The results showed a normative value of 36.6 sec in the control and 67.2 sec in the hemophilic group. Statistical analysis of the data showed that the walking ability was significantly reduced in the hemophilic group. These normative values illustrate a useful, simple, reproducible, rapid assessment of walking disability in adults with hemophilic arthropathy, and also aid the planning of treatment.

## Introduction

A lack of factor VIII (FVIII) or factor IX (FIX) results in hemophilia, a blood-clotting illness.^1^ The mode of inheritance is chromosome X-linked and recessive. Accordingly, grandfathers pass the illness to 50% of their grandchildren through their daughters (who are known carriers but do not have the illness). There are, nevertheless, sporadic spontaneous mutations.^2^ Depending on the amount of the blood-clotting factor, there are different categories: mild hemophilia (>5–40% FVIII/FIX), which causes bleeding during surgical procedures or from severe injuries, and severe hemophilia (1% FVIII/IX),.^3^ The primary symptom of the latter form is spontaneous and recurrent bleeding into joints, muscles, and soft tissues, with the first joint bleed typically occurring in the first few years of life. Unpreventable bleeding may cause arthropathy, chronic discomfort, and muscular atrophy, which in turn results in incapacity.^4^ Hemorrhages affect the musculoskeletal system (80% of cases), the central nervous system in 20%, and other organ systems in 2%.^5^ The joint becomes inflamed, stiff and warm after intraarticular bleeding, and the skin turns red. The patient feels severe pain and holds the injured joint in flexion to dull it. Severe discomfort, decreased range of motion (ROM) of the affected joints, a loss of joint functions, and incapacity are all brought on by recurrent intraarticular hemorrhage episodes.^6^ In addition, intraarticular hemorrhage causes synovial inflammation and blood-related cartilage damage, and recurrent joint bleeding leads to hemophilic arthropathy, which results in degenerative cartilage and bone abnormalities.^7^ Deformities including restricted motion, a valgus hindfoot, and altered posture are brought on by the advancement of hemophilic arthropathy and the degradation of the joint. Plano-valgus foot or subtalar and tibiofibular joints may happen. In patients with severe hemophilia, prolonged pain brought on by hemophilic arthropathy is a reliable indicator of impairment.^1^ This has restrictions on joint function, activity performance, and social participation over the short- and long-term.^5^ The conventional treatment for hemophilia entails surgical intervention to enhance joint function, when necessary, as well as managing or avoiding bleeding with a specific coagulation factor.^8^

The aim of this study was to determine the walking ability of severe hemophilic patients, as compared to the normal population, as measured by a 50-m walk test time.

## Materials and Methods

This was a cross-sectional study carried out at the Hemophilia Daycare Centre, PGIMS Rohtak, India, for which ethical clearance was granted by the Ethical Committee of Pt. B. D. Sharma, University of Health Sciences, Rohtak vide letter no. BREC/23/017. The study was performed in accordance with the ethical standards as laid down in the 1964 Declaration of Helsinki. Sixty male subjects aged 18-30 years were selected into 2 groups, 30 in hemophilic and 30 in normal control group. Only patients with severe hemophilia A & B and knee arthropathy were included. Participants with neurological illness, fractures, or psychological conditions that might interfere with the test were excluded from the study.

The 50-meter walk test consisted of a patient or normal control walking non-stop 2.5 times between two cones positioned 10 meters apart along a 50-meter walking course. The circuit was circular in which 2 cones were placed at 10 meters distance which indicated where to change the direction. They have to walk the 10-meter track 2.5 times around, without stopping. The interval timings were recorded using the stopwatch, which ran from the beginning of the walk to 40 m (the second return trip), pressing the stop button at 50 m. The time needed to walk 50 metres and the times needed to walk each of laps 1–5 were the assessment items that were recorded (all timings were taken in seconds). Measurements were performed twice. The interval between one cone and the other, which was 10 m apart, was classified as one lap.^9^

The SPSS statistical package (version 25.0) was used to analyze the data. Descriptive statistics were used to calculate the mean and standard deviation of the time taken to complete the 50-meter walk test. Student-independent t-test was used to find any difference between the hemophilic and control groups. For all statistical tests, a p-value ≤ 0.05 was taken as a significant difference.

## RESULTS

**Figure 1.**
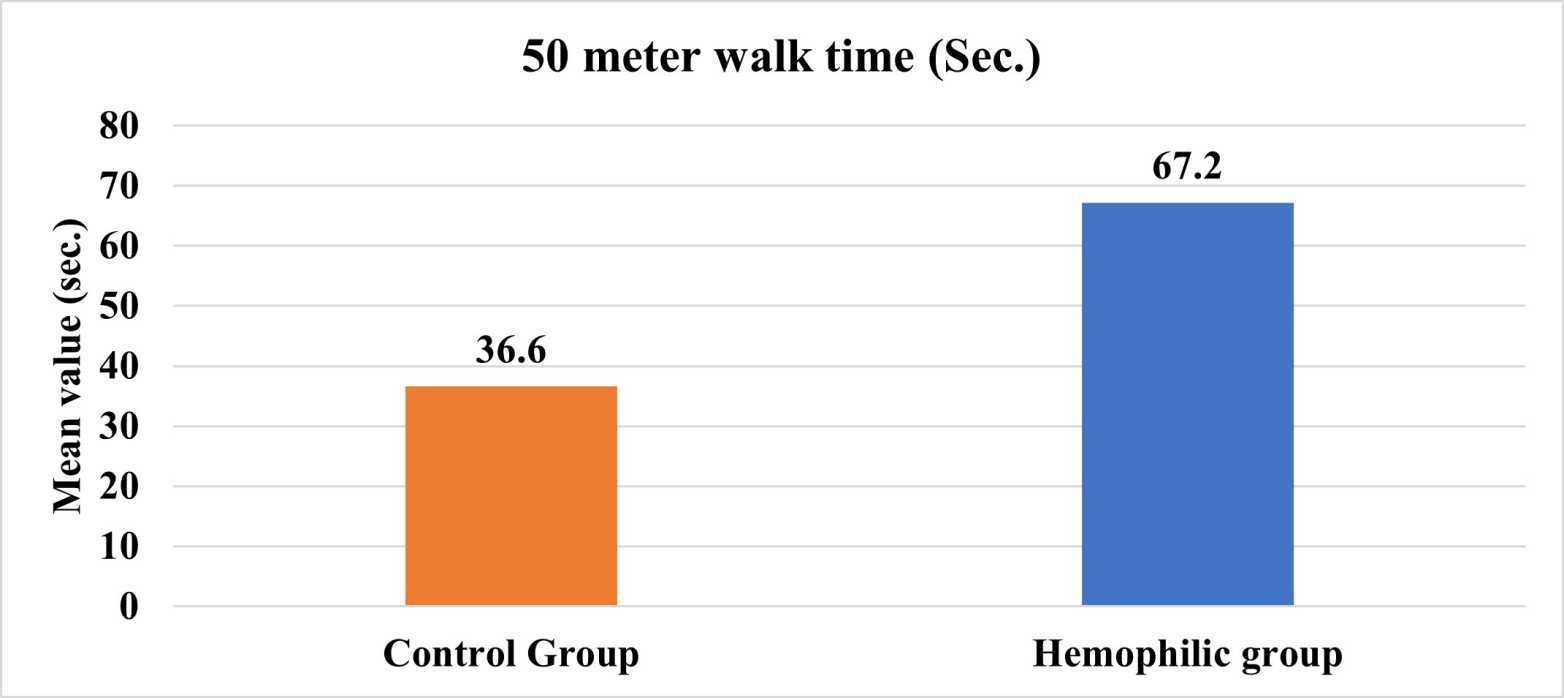
Comparison of the 50- meter walk test time between the control and the hemophilic group

The mean age was 27.48±5.35 years in the control group and 26.76±4.84 years in the hemophilic group. Table 1 shows the mean±SD values of the 50- meter walk test in the two groups. The difference in these values was significant (p≤0.05). Figure 1 shows the data as bar graphs.

**Table 1.** Comparison of the walking ability of the control versus the hemophilic group using independent t-test

**Walking ability**	**Control group**		**Hemophilic group**		**t-value**	**p-value**
	**Mean**	**SD**	**Mean**	**SD**		
**50-meter walk test**	36.6	4.02	67.2	15.5	10.3	<0.05**

* Significant difference at 0.05 level

## DISCUSSION

There is a scarcity of data on the walking ability of patients with hemophilia. Previous reports have focused on gait analysis,^10^ functional abilities,^11^ joint health,^12^ aerobic capacity,^13^ quality of life,^14^ and changes in adaptive, emotional, and behavioral functioning in children and adolescents with hemophilia.^15^ Therefore, this study was carried out to find the normative value of a 50- meter walk test in the hemophilic and the normal populations. It showed a significant difference in the walking abilities between the control and the hemophilic group. This difference can be due to knee arthropathy present in patients with hemophilia which disturbs walking and makes the patient slow down and change their gait pattern. As such, the average time for the 50-meter walk in the hemophilic group was 83.61% longer than that in the normal group. These results are consistent with a previous study by Radzevic et al. (2013) in which they found Lithuanian children with hemophilia showed reduced physical activity and functional ability when compared with their healthy peers.^16^

In conclusion, establishing the normative value of a 50- meter walk test of patients with hemophilia may be useful for planning treatment and assessing its efficacy upon follow-up. Future studies should include larger sample sizes and additional variables for assessing walking abilities.
